# A prospective study of non-invasive prenatal screening technology in preimplantation genetic testing cycles

**DOI:** 10.3389/fmed.2026.1880334

**Published:** 2026-08-12

**Authors:** Hongwei Yan, Yun Wang, Mingjia Zhao, Rong Li, Jin Huang

**Affiliations:** 1 Department of Obstetrics and Gynecology, Center for Reproductive Medicine, Peking University Third Hospital, Beijing, China; 2 Department of Reproduction and Genetics, Maternity and Child Health Care Hospital of Tangshan, Tangshan, Hebei, China; 3 National Key Laboratory for the Promotion of Female Fertility, Beijing, China; 4 National Clinical Medical Research Center for Gynecological and Obstetric Diseases, Peking University Third Hospital, Beijing, China; 5 Key Laboratory of the Ministry of Education for Assisted Reproduction, Peking University, Beijing, China; 6 Beijing Key Laboratory of Collaborative Innovation in Frontier Technologies for Population Quality, Beijing, China

**Keywords:** *in vitro* fertilization and embryo transfer, invasive pranatal testing, non-invasive prenatal testing, preimplantation genetic testing, prenatal diagnosis

## Abstract

**Objective:**

To investigate the role of NIPT 2.0 in prenatal screening among populations undergoing PGT.

**Methods:**

This study enrolled 113 patients who underwent NIPT 2.0 after 12 weeks of gestation at the Reproductive Medicine Center of Peking University Third Hospital between January 2024 and July 2025. Patients were categorized into PGT (*n* = 55), IVF (*n* = 23), and spontaneous pregnancy (*n* = 35) groups. Amniocentesis performed based on genetic counseling and obstetric indications. Perinatal outcomes were compared across groups and within PGT subgroups.

**Results:**

56 women underwent both NIPT 2.0 and IPT, with 100% concordance between tests. NIPT 2.0 results indicated low fetal genetic risk in all 113 pregnant women. Among these, 56 underwent IPT: (37 PGT,9 IVF,10 spontaneous pregnancy group), All 56 prenatal diagnosis results were negative. There were no statistically significant differences among the three groups in miscarriage rate, live birth rate, preterm birth rate, birth defect rate, or neonatal birth weight and length. Further subgroup analysis of PGT Groups showed no significant differences in pregnancy outcomes between patients who underwent IPT and those who did not.

**Conclusion:**

NIPT 2.0 can serve as a prenatal screening method following PGT-assisted pregnancies. Whether it can replace invasive prenatal testing requires further in-depth research.

## 1 Introduction

Preimplantation genetic testing (PGT) has become an essential tool in assisted reproductive technologies for screening embryos for aneuploidy (PGT-A), structural rearrangements (PGT-SR), and monogenic disorders (PGT-M). Its application aims to improve clinical pregnancy outcomes and reduce the risk of birth defects caused by genetic abnormalities (1, 2). However, PGT is performed on a limited number of trophectoderm cells, which may not invariably represent the genetic constitution of the inner cell mass or fetus because of mosaicism, sampling variability, and technical error (3–5). Consequently, risks of false negatives or positives exist because of technical limitations and embryonic mosaicism. For these reasons, prenatal testing is generally recommended for patients undergoing PGT who achieve successful implantation and pregnancy (6).

Traditional invasive prenatal testing (IPT) includes chorionic villus sampling (CVS) in early pregnancy, amniocentesis in mid-pregnancy, and cordocentesis, with amniocentesis being the most widely used and considered the “gold standard” for fetal prenatal diagnosis (7). However, these invasive procedures carry potential risks of infection and miscarriage (8). Non-invasive prenatal testing (NIPT) emerged following the discovery of cell-free fetal DNA (cffDNA) in maternal peripheral blood plasma in 1997 (9). By analyzing cffDNA in maternal plasma, NIPT demonstrated high sensitivity and specificity for common chromosomal aneuploidies, and is gradually becoming a widely accepted prenatal screening method (10, 11). Next-generation NIPT technology (referred to as “NIPT 2.0”) enables simultaneous non-invasive prenatal screening for chromosomal euploidy, microdeletions/microduplications, and select monogenic disorders. This expands the potential screening scope of NIPT; however, evidence regarding its clinical performance and utility, particularly in post-PGT pregnancies, is still evolving (12–14).

High-level evidence remains limited regarding the optimal prenatal testing strategy after PGT. Previous studies suggest that NIPT may be a reasonable screening option after PGT-A and, in selected cases, PGT-SR, whereas its role after PGT-M remains limited; conventional prenatal screening may also perform differently in PGT-A pregnancies (15, 16). Professional recommendations similarly vary, ranging from acceptance of cfDNA-based screening after euploid embryo transfer to a more cautious, individualized approach based on the PGT indication, residual genetic risk, and need for diagnostic confirmation (17, 18).

We hypothesized that, among participants who underwent both tests, NIPT 2.0 would show a high level of concordance with invasive prenatal testing and that the evaluated pregnancy and neonatal outcomes would be broadly comparable across the PGT, conventional IVF, and spontaneous pregnancy groups. Accordingly, the primary outcome was the concordance between NIPT 2.0 and invasive prenatal testing in the paired-testing subset. Secondary outcomes included miscarriage, live birth, preterm birth, fetal or birth defects, neonatal birth weight and length, mode of delivery, and the uptake of invasive prenatal testing across the three conception groups. Differences according to PGT indication were assessed as exploratory subgroup outcomes.

## 2 Materials and methods

### 2.1 Study design and ethics

This was a prospective, single-center, observational cohort study approved by the Ethics Committee of Peking University Third Hospital (2023 Medical Ethics Review No. 665–01). All participating couples signed informed consent forms prior to enrollment.

### 2.2 Study population

**FIGURE 1:**
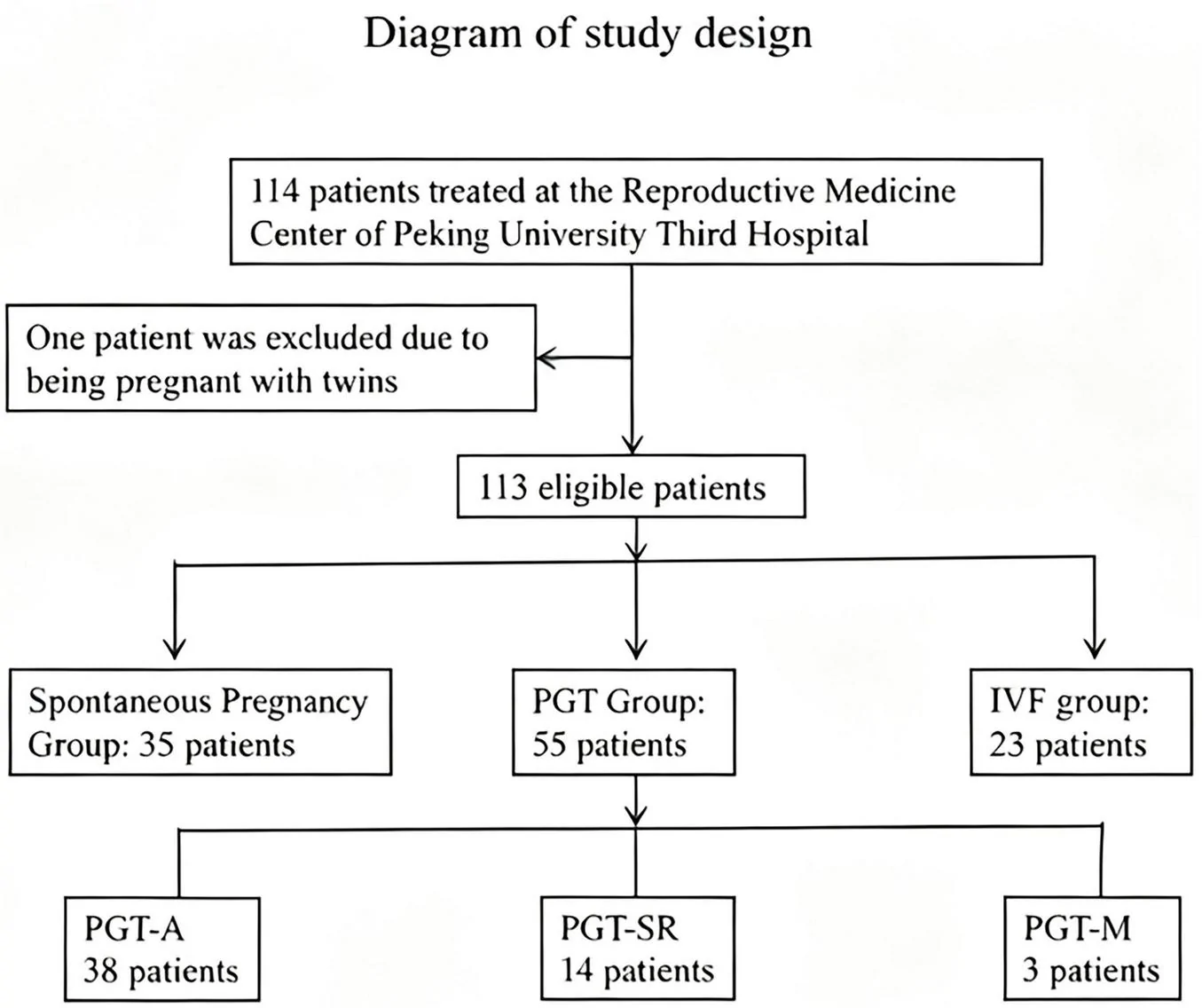
Patients classified according to the techniques used for testing during pregnancy. IVF, *in vitro* fertilization; PGT, preimplantation genetic testing; PGT-A, PGT-aneuploidy; PGT-M, PGT-monogenic disorders; PGT-SR, PGT-structural rearrangement.

This study included 113 pregnant women who underwent NIPT 2.0 at the hospital between January 1, 2024, and July 31, 2025, and completed follow-up through March 31, 2026; no additional targeted genetic testing or genetic evaluation was performed during the follow-up period. Participants were divided into three groups based on conception method: PGT group (Group A, *n* = 55): Pregnancy achieved after transferring a PGT-screened euploid or unaffected single blastocyst, *in vitro* fertilization (IVF) group (Group B, *n* = 23): Pregnancies achieved through assisted reproductive technology without PGT, Spontaneous pregnancy group (Group C, *n* = 35): Pregnancies occurred spontaneously. The PGT group was further subdivided into three subgroups based on indications: PGT-A (Group A1): 38 couples underwent embryo aneuploidy testing because of recurrent miscarriage, repeated implantation failure, or similar factors. PGT-SR (Group A2): 14 couples underwent embryo aneuploidy testing because of chromosomal structural rearrangements in one partner; PGT-M (Group A3): three couples underwent embryo genetic testing because of polycystic kidney disease in one partner. Exclusion criteria included: multiple pregnancies, miscarriage before 12 weeks gestation, incomplete data, or severe maternal comorbidities. The study design is detailed in Figure 1.

### 2.3 Data collection

Baseline data were collected from electronic medical records: age, body mass index, baseline serum hormone levels [anti-Mullerian hormone (AMH), follicle-stimulating hormone (FSH), luteinizing hormone (LH)], obstetric history, history of recurrent miscarriage, endometrial thickness (mm), gonadotropin (Gn) stimulation dose (IU), Gn stimulation duration (days), proportion of primary infertility cases; pregnancy outcome measures: miscarriage (clinical pregnancy loss before 28 weeks gestation), preterm birth (delivery before 37 weeks gestation), live birth (delivery of a viable infant after 22 weeks gestation), birth defects (anatomical, functional, or metabolic abnormalities present before or after delivery), neonatal birth weight and length, gestational age at delivery, mode of delivery; prenatal testing information: type of testing received (NIPT and/or amniocentesis) and the results.

### 2.4 Embryo culture, PGT procedures, embryo transfer, and pregnancy outcome assessment in PGT and IVF groups

Ovarian stimulation for patients in both the PGT and IVF groups followed our center’s standard protocol. When at least two follicles reached 18 mm in diameter, trigger day involved administration of recombinant human chorionic gonadotropin (hCG) or urinary hCG to induce final maturation of preovulatory primary oocytes. Oocyte retrieval was subsequently performed under transvaginal ultrasound guidance 34–38 h later. Fertilization was achieved using either IVF or intracytoplasmic sperm injection as indicated. Fertilization status was assessed 16–18 h post-insemination (day 1). Patients in the IVF group had the option of day 3 or 5 embryo transfer, or embryo transfer during a thaw cycle. For the PGT group, embryos with ≥ 4 cells and a fragmentation rate < 50% (excluding embryos originating from multinucleated embryos) were selected on day 3 for blastocyst culture. In all PGT cycles, blastocysts graded 4BC or higher underwent trophoblast biopsy (19), with biopsied cells subjected to next generation sequencing testing after whole-genome amplification. Following biopsy, all blastocysts underwent vitrification cryopreservation as single blastocysts. Thawing and transfer were performed according to the center’s routine protocol using either a natural or artificial cycle. Each transfer cycle involved a single blastocyst transfer, with embryos transferred in PGT cycles being those showing no genetic abnormalities. If all blastocysts obtained from a single stimulation cycle were diagnosed as abnormal, that cycle was canceled. Patients in both the IVF and PGT groups were considered pregnant if serum β-hCG ≥ 25 U/L was detected 2 weeks post-transfer. Clinical pregnancy was defined as the observation of a gestational sac and fetal heartbeat via ultrasound approximately 30–40 days post-transfer. Those achieving clinical pregnancy underwent follow-up to document pregnancy and delivery outcomes.

### 2.5 Assessment of pregnancy outcomes in the spontaneous pregnancy group

Patients with spontaneous pregnancy were defined as clinically pregnant when a gestational sac and fetal heartbeat were observed via ultrasound around 6 weeks of gestation. Those achieving clinical pregnancy were followed up, with pregnancy and delivery outcomes recorded.

### 2.6 Prenatal testing strategy

All enrolled pregnant women underwent NIPT 2.0 testing after 12 weeks of gestation. It was operationally defined as an expanded maternal plasma cell-free DNA-based prenatal screening assay using unique molecular identifier (UMI)-mediated error suppression, hybridization-based target enrichment, and high-depth targeted next-generation sequencing to detect *de novo* or paternally inherited pathogenic/likely pathogenic fetal variants in 30 genes associated with dominant monogenic disorders. Sequencing was performed on an Illumina HiSeq 2500 platform using 2 × 100-bp paired-end reads. Results were reported only for samples with a fetal fraction of ≥ 4.5% and a minimum sequencing depth of 200 × across ≥ 97% of the targeted regions. Variants with a variant allele fraction of ≥ 0.5% entered the initial analysis, and candidate fetal variants were additionally required to have a Z score of > −0.6. All positive findings were confirmed using an independent amplicon-based assay on plasma cfDNA and interpreted together with maternal leukocyte and parental genomic DNA to determine variant origin. Sensitivity and specificity were defined as true positives/(true positives + false negatives) and true negatives/(true negatives + false positives), respectively, using invasive prenatal testing, postnatal genetic testing, or clinical follow-up as the reference standard; exact binomial 95% confidence intervals were calculated. The assay was primarily intended for *de novo* or paternally inherited sequence variants within the targeted panel, and its performance should not be extrapolated to genes outside the panel, maternally inherited variants, complex structural variants, or other unvalidated variant classes (12).

Among the 56 participants included in the paired-testing analysis, invasive prenatal diagnosis was performed by amniocentesis, and all amniotic-fluid samples underwent conventional karyotyping and chromosomal copy-number variation analysis. Any pathogenic or likely pathogenic variant detected by the primary NIPT 2.0 assay would be confirmed using an independent amplicon-based NGS assay on maternal plasma cfDNA, with parental leukocyte DNA analyzed to determine variant origin. Sanger sequencing of an invasive fetal or postnatal sample was planned when such a sample was available.

In addition, women in the PGT group were fully informed about the limitations of PGT and the diagnostic value of invasive procedures. For the IVF and spontaneous pregnancy groups, obstetricians recommended invasive prenatal testing based on individual circumstances. All patients provided informed consent and independently chose whether to undergo amniocentesis for prenatal testing. This study did not influence clinical decisions and documented the final prenatal testing pathway selected.

### 2.7 Statistical analysis

Statistical analyses were performed using SPSS version 27.0. Continuous variables are presented as mean ± standard deviation and were compared across groups using one-way analysis of variance; categorical variables are presented as frequency (percentage) and were compared using the chi-square test or Fisher’s exact test, as appropriate. All tests were two-sided, and *P* < 0.05 was considered statistically significant. No formal a priori sample-size calculation was performed because this exploratory prospective cohort included all eligible participants recruited during a predefined study period. The study was not designed or powered to assess diagnostic accuracy, equivalence, or non-inferiority; therefore, concordance findings and subgroup analyses were interpreted descriptively and exploratorily.

## 3 Results

### 3.1 General characteristics of the study population

A total of 113 patients were included in this study, comprising 55 in the PGT group (Group A), 23 in the IVF group (Group B), and 35 in the spontaneous pregnancy group (Group C). Significant differences existed among the three groups in age (*P* = 0.017), endometrial thickness (P = 0.005), number of pregnancies (*P* < 0.001), and proportion of recurrent miscarriages (*P* < 0.01). Specifically, the PGT group had a higher age and more pregnancies, while the IVF group exhibited a thicker endometrium. No significant differences were observed among groups for the following parameters: AMH, basal FSH and LH, Gn stimulation dose, Gn stimulation duration, or proportion with primary infertility (Table 1).

**TABLE 1:** Comparison of general characteristics among three patient groups.

Parameters	Group A (*n* = 55)	Group B (*n* = 23)	Group C (*n* = 35)	*P*
Age (years)	36.09 ± 4.34	35.87 ± 3.65	33.71 ± 3.34	0.017*
BMI (kg/m2)	24.03 ± 3.28	22.36 ± 2.39	22.89 ± 3.40	0.068
Basal FSH (mIU/mL)	6.96 ± 3.08	6.93 ± 2.21	–	0.969
Basal LH (mIU/mL)	3.91 ± 1.92	4.91 ± 2.28	–	0.054
AMH (ng/ml)	3.17 ± 2.06	4.25 ± 4.14	–	0.132
Endometrial thickness (mm)	9.64 ± 1.65	10.82 ± 1.53	–	0.005*
Gn Stimulation Dose(IU)	2676.81 ± 958.56	2583.96 ± 1122.92	–	0.716
Gn stimulation duration(days)	10.00 ± 1.61	10.45 ± 1.71	–	0.275
Primary infertility prevalence				0.795
Yes	4(40.0%)	2(20.0%)	4 (40%)	
No	51(49.5%)	21(20.4%)	31(30.1%)	
Number of pregnancies				<0.001*
<3 times	23(34.3%)	19(28.4%)	25(37.3%)	
≥ 3 times	32(69.6%)	4(8.7%)	10(21.7%)	
Prevalence of recurrent miscarriage %(n)	85.0(34/55)	0	17.1(6/55)	<0.01*

Group A; pregnancy achieved after blastocyst screening by preimplantation genetic testing. Group B; pregnancy achieved after *in vitro* fertilization. Group C; pregnancy achieved after natural conception. AMH; anti-Mullerian hormone, BMI; body mass index, FSH; follicle-stimulating hormone, Gn; gonadotropin, LH; luteinizing hormone. There was a statistically significant difference in age between the AC and BC groups (*P* < 0.05), but no statistically significant difference between the AB groups (*P* = 0.82).

*Represents statistically significant difference (*P* < 0.05).

### 3.2 Comparison of NIPT 2.0 results with IPT findings

Among 113 patients, 56 underwent both NIPT 2.0 and amniocentesis for fetal genetic testing. This included 37 cases in the PGT group (22 PGT-A, 12 PGT-SR, three PGT-M), nine in IVF group, and 10 in the spontaneous pregnancy group. The NIPT 2.0 results for all 56 patients indicated no fetal abnormalities, specifically: no detection of common chromosomal aneuploidies (e.g., trisomies 21, 18, and 13), no clinically significant chromosomal microdeletion/microduplication syndromes, and negative results for targeted monogenic disorders (e.g., polycystic kidney disease, neurofibromatosis). Concurrent IPT (combining karyotyping with chromosomal copy number variation analysis) yielded results fully consistent with NIPT 2.0, with no evidence of fetal genetic abnormalities. Postnatal measurements including birth weight and length fell within normal ranges.

### 3.3 Comparison of clinical pregnancy outcomes and prenatal screening methods

There were no statistically significant differences among the three groups in primary clinical outcome measures (miscarriage, live birth, preterm birth, and birth defect rates) or in neonatal birth weight and length (*P* > 0.05). Regarding prenatal testing methods, the proportion of patients undergoing amniocentesis was significantly higher in the PGT group than in the IVF and spontaneous pregnancy groups (*P* < 0.001) (Table 2). Among all patients, two cases of fetal abnormalities occurred: one PGT-A patient diagnosed with Beckwith-Wiedemann syndrome at 24 weeks gestation via ultrasound, followed by termination after genetic confirmation via cordocentesis; another natural pregnancy terminated at 24 weeks because of severe fetal malformation (cleft lip and palate). Regarding delivery methods, the cesarean section rate was significantly higher in Group A than in the other two groups (*P* = 0.044).

**TABLE 2:** Comparison of clinical pregnancy outcomes among three patient groups and prenatal screening methods.

Parameters	Group A (*n* = 55)	Group B (*n* = 23)	Group C (*n* = 35)	*P*
Miscarriage rate %(n)	3.6(2/55)	4.3(1/23)	2.9(1/35)	0.954
Preterm birth rate %(n)	14.5(8/55)	4.3(1/23)	8.6(3/35)	0.337
Live birth rate %(n)	94.5(52/55)	95.7(22/23)	94.3(33/35)	0.971
Fetal defect rate %(n)	1.8(1/55)	0	2.9(1/35)	0.600
Birth weight of newborns (kg)	3.11 ± 0.84	3.46 ± 0.36	3.34 ± 0.83	0.149
Newborn length at birth (cm)	48.26 ± 4.65	49.77 ± 1.63	49.09 ± 3.95	0.302
Mode of delivery				0.044*
natural childbirth %(n)	39.6(21/53)	68.2(15/22)	58.8(20/34)	
Cesarean section %(n)	60.4(32/53)	31.8(7/22)	41.2(14/34)	
Amniocentesis rate%(n)	67.3(37/55)	39.1(9/23)	28.6(10/35)	<0.001*

Group A, pregnancy achieved after blastocyst screening by preimplantation genetic testing; Group B, pregnancy achieved after *in vitro* fertilization; Group C, pregnancy achieved after natural conception.

*Represents statistically significant difference (*P* < 0.05).

### 3.4 Comparison of pregnancy outcomes and prenatal testing method selection in PGT subgroups

Subgroup analysis was conducted within the PGT group based on indications (PGT-A group as A1, PGT-SR as A2, PGT-M as A3). No significant differences were observed among groups in miscarriage rate, preterm birth rate, live birth rate, fetal anomaly rate, or neonatal weight and length (*P* > 0.05). However, amniocentesis rates differed significantly between indication groups (*P* = 0.042). The PGT-M group had three cases, all undergoing PGT for polycystic kidney disease, with all three patients receiving amniocentesis. The PGT-SR group had the next highest rate, while the PGT-A group had the lowest rate (Table 3).

**TABLE 3:** Comparison of pregnancy outcomes in preimplantation genetic testing (PGT)-assisted subgroups.

Parameters	Group A1 (*n* = 38)	Group A2 (*n* = 14)	Group A3 (*n* = 3)	*P*
Miscarriage rate %(n)	5.3(2/38)	0	0	0.469
Preterm birth rate %(n)	15.8(6/38)	7.1(1/14)	33.3(1/3)	0.484
Live birth rate %(n)	92.1(35/38)	100(14/14)	100(3/3)	0.317
Fetal defect rate %(n)	2.6(1/38)	0	0	0.688
Birth weight of newborns (kg)	3.14 ± 0.81	3.11 ± 0.74	2.71 ± 1.75	0.709
Newborn length at birth (cm)	48.44 ± 4.09	48.50 ± 4.97	45.00 ± 9.64	0.466
Amniocentesis rate %(n)	57.9(22/38)	85.7(12/14)	100(3/3)	0.042

Group A1, PGT-aneuploidy testing; Group A2, PGT-structural rearrangement testing; Group A3, PGT-monogenic disorders testing.

## 4 Discussion

Prenatal screening and diagnosis can effectively reduce the incidence of severe birth defects in newborns, decrease the consumption of public healthcare resources such as neonatal intensive care and disability rehabilitation, and improve the quality of the newborn population. Since its clinical translation, NIPT has become the most widely used prenatal genetic screening method globally (20–22). The American College of Medical Genetics and Genomics explicitly recommends NIPT as the first-line approach for screening fetal chromosomal abnormalities in low-risk populations in its latest clinical guidelines (10).

NIPT performs genetic analysis by sequencing placental-derived cffDNA in maternal plasma. Constrained by its “placental origin” nature and narrow detection range (limited to trisomies 21, 18, and 13), traditional testing is susceptible to interference from mosaicism or maternal variants, making it difficult to meet the screening needs of PGT populations for microdeletions and single-gene disorders (23, 24). NIPT 2.0 employs targeted capture and high-depth sequencing of cffDNA, enhancing accuracy for chromosomal aneuploidy and microdeletion/microduplication detection while expanding screening coverage to select autosomal dominant monogenic disorders (25, 26). Results from a retrospective clinical study demonstrated that NIPT 2.0 had a sensitivity of 100% and a specificity of 99.3% (27).

PGT is a fertility assistance method for specific populations at genetic risk, making post-conception fetal genetic testing equally critical. PGT fundamentally involves embryo genetic screening rather than definitive fetal diagnosis. Additionally, chorionic villus sampling carries risks of mosaicism, and the limited number of cells collected may not fully represent the fetal genetic state (28, 29). The American Society for Reproductive Medicine (2024) indicated that PGT-A carries a 1–2% risk of misdiagnosis and de novo aneuploidy, while PGT-M faces challenges related to allelic dropout (30, 31). The 2025 Italian Society of Human Genetics guidelines emphasize that IPT remains the “gold standard” for validating PGT-SR/M (18).

In this study, 56 pregnant women underwent both NIPT 2.0 and IPT, with both tests yielding identical results that did not indicate fetal genetic abnormalities. This outcome provides further evidence supporting the use of NIPT 2.0 as a fetal genetic screening method for individuals who have undergone PGT. Previous studies have shown favorable analytical performance for selected abnormalities in defined populations, and our findings provide additional preliminary evidence supporting the feasibility of NIPT 2.0 after PGT (32, 33). However, its diagnostic performance in post-PGT pregnancies and its applicability beyond the validated assay scope remain uncertain. In the current study, the 37 cases in the PGT group included 22, 12, and three patients in the PGT-A, PGT-SR, and PGT-M groups, respectively. Previous studies suggest that non-invasive screening may reduce concerns regarding invasive procedures while providing a preliminary, non-diagnostic risk assessment before further evaluation (18, 34). Compared with the conventional IVF group (9 cases) and spontaneous pregnancy group (10 cases), the consistency between negative NIPT 2.0 results and normal neonatal growth indicators (e.g., weight and height) in the PGT group further underscores its screening value in high-risk populations (35, 36). Therefore, the integrated application of NIPT 2.0 helps address these shortcomings, particularly in the PGT-M and PGT-SR subgroups, where it can provide supplementary screening for monogenic disorders and structural rearrangements (18, 37). In summary, as an earlier prenatal diagnostic technique, PGT warrants attention for both prenatal screening and diagnosis during pregnancy. Based on different PGT indications and the pregnant woman’s condition, appropriate prenatal screening and diagnostic methods should be selected to reduce residual risks after PGT and lower the incidence of birth defects in newborns. With the advancement of non-invasive prenatal screening and genetic testing technologies, the use of fetal cffDNA in maternal blood to validate PGT accuracy will be further developed and refined in the future (38, 39).

Subgroup analysis of PGT groups based on indications revealed no significant differences among the three groups in miscarriage rates, preterm birth rates, live birth rates, birth defect rates, or neonatal weight and length. No significant differences in the evaluated perinatal outcomes were observed among the PGT subgroups; however, the small subgroup sizes preclude definitive conclusions. Furthermore, all PGT cycles employed single embryo transfer, thereby reducing obstetric risks associated with multiple pregnancies. A 2024 retrospective study by Liang et al. demonstrated no significant differences in miscarriage, live birth, preterm birth, or birth defect rates among patients undergoing NIPT or IPT post-PGT across different indication subgroups, supporting the comparability of NIPT outcomes in PGT pregnancies (15). Similarly, in 2018, Kimelman et al. reported that patients undergoing PGT-A predominantly opted for NIPT, yielding outcomes comparable with invasive testing (34). In this study, the proportion of patients undergoing IPT differed significantly across the three subgroups. Amniocentesis was performed in all cases in the PGT-M group, with the next highest rate of testing in cases in the PGT-SR group, and the lowest rate in cases in the PGT-A group. This variation primarily stems from differing genetic risk levels associated with each PGT indication, technical limitations, and varying clinical guideline requirements for prenatal confirmation. Patients in the PGT-M group exhibited the highest demand for definitive diagnosis of monogenic disorders. This stems from the risk of misdiagnosis inherent in PGT-M technology (e.g., allele loss, amplification failure) and the current limitations of NIPT in detecting monogenic diseases, which cannot fully replace invasive diagnosis (30). In this study, all three cases in the PGT-M group involved polycystic kidney disease, and amniocentesis confirmed the absence of genetic risk in the fetuses. Given the very small PGT-M subgroup (*n* = 3), its results are descriptive and exploratory only; no conclusion regarding test performance in PGT-M pregnancies can be drawn. The PGT-SR group involved structural abnormalities like balanced translocations, carrying higher residual unbalanced risk, hence higher rates of IPT acceptance among couples (18). The PGT-A group primarily screens for aneuploidy, where NIPT demonstrates high sensitivity for common aneuploidies and lower post-PGT-A risk, leading patients to prefer non-invasive NIPT (6). These subgroup findings further confirm that among successful PGT-assisted pregnancies, primary perinatal outcomes are comparable across different indications. This provides evidence supporting NIPT as a reliable screening approach for PGT-A and certain patients with PGT-SR. However, for PGT-M, invasive diagnosis remains indispensable.

In this study, significant differences among the three patient groups in age, endometrial thickness, number of pregnancies, and obstetric history primarily stem from variations in patient demographics, clinical indications, and embryo transfer protocols associated with different conception methods (40, 41). No significant differences were observed among the three groups in AMH, baseline FSH and LH, Gn stimulation dose, Gn stimulation duration, or the proportion of primary infertility cases. This is primarily because these parameters reflect ovarian reserve and stimulation response rather than being directly influenced by conception method or PGT indications (42, 43). These equivalent parameters between the groups reflect the effectiveness of personalized clinical management, enhance the comparability between groups in this study, and further support conclusions regarding primary clinical outcomes (perinatal indicators). Although baseline characteristics in the PGT group (e.g., advanced maternal age, complex obstetric history) are typically associated with higher perinatal risks, this study found no significant differences among the three groups in primary pregnancy outcomes. This may be because PGT screening and transferring euploid blastocysts reduced the risk of adverse outcomes caused by chromosomal abnormalities, making PGT pregnancy outcomes comparable with or equivalent to conventional IVF and spontaneous pregnancy (44).

Because no abnormal NIPT 2.0 or invasive diagnostic results were observed, sensitivity, specificity, predictive values, and false-negative rates could not be estimated. The findings therefore demonstrate only concordant negative results in the paired-testing subset and should not be interpreted as evidence of diagnostic equivalence. This prospective cohort provides preliminary evidence for the feasibility of NIPT 2.0 after PGT; however, its interpretation is limited by the small sample size, single-center design, and non-random selection of prenatal testing, as all participants underwent NIPT whereas IPT was selected voluntarily after counseling and according to perceived risk. Longitudinal follow-up is ongoing to document delivery outcomes, neonatal health, and the subsequent growth and developmental trajectories of the offspring. NIPT 2.0 should therefore be incorporated into a structured pre- and post-test counseling pathway rather than presented as a replacement for invasive prenatal diagnosis, particularly when definitive confirmation is clinically indicated. Its health-economic value also remains uncertain and requires prospective cost-effectiveness evaluation. Overall, NIPT 2.0 may represent a less invasive and more acceptable screening option after PGT, while appropriately selected patients should continue to be offered diagnostic testing. Larger multicenter studies including abnormal cases and longer-term offspring follow-up are required to establish its diagnostic performance, clinical utility, and optimal role in post-PGT prenatal care.

## Data Availability

The original contributions presented in this study are included in the article/supplementary material, further inquiries can be directed to the corresponding author.
